# Feasibility and acceptability of a community health worker administered behavioral activation intervention for postpartum depression: a single arm pilot study from India

**DOI:** 10.3389/fpsyt.2024.1284674

**Published:** 2024-04-29

**Authors:** Amy Szajna, Bheemsain Tekkalaki, Veereshkumar Nandagaon, Gururaj Udapi, Manjunath Sogalad, Shweta Dandagi, Uma Kole, Sushma Patil, Sudha Raddi, Vanessa Short, Patricia J. Kelly

**Affiliations:** ^1^ College of Nursing, Thomas Jefferson University, Philadelphia, PA, United States; ^2^ Jawaharlal Nehru Medical College, Belgaum, Karnataka, India; ^3^ College of Nursing, KLE University, Belgaum, Karnataka, India; ^4^ College of Applied Medical Sciences Department of Nursing University of Bisha, Bisha, Saudi Arabia

**Keywords:** postpartum depression, South India, behavioral activation, community-health worker, maternal mental health

## Abstract

**Introduction:**

Women in India experience high rates of postpartum depression (PPD), with minimal availability of screening or treatment. India has an extensive network of community health workers, known as accredited social health activists (ASHAs). While they are knowledgeable about most maternal–child health problems, they have minimal knowledge about PPD. We trained ASHAs to deliver a simple home-based intervention, behavioral activation (BA), which involves individuals in activities that are sources of positive reinforcement to counter depression. The research questions guiding this study were as follows: 1) What are the feasibility and acceptability of ASHAs screening for and delivering a brief behavioral activation intervention addressing PPD among women in Belagavi, South India? 2) What impact did the brief behavioral activation intervention have on PPD?

**Methods:**

The mixed methods evaluation used interviews with participants and interventionists, and depression scores were assessed before and after the evaluation. After a 2-day training with 17 ASHAs that focused on understanding PPD, screening using the Edinburgh Postnatal Depression Scale (EPDS), and implementing the BA protocol, ASHAs and researcher supervisors screened the mothers 6–12 weeks postpartum presenting at pediatric immunization clinics. Mothers who screened positive were invited to participate in an ASHA-led 5-week BA intervention, with ASHAs visiting the mothers’ homes. We assessed post-intervention EPDS scores and conducted satisfaction assessments and individual interviews.

**Results:**

All 26 women who screened positive on the EPDS agreed to be enrolled in the study. All participants had a significant reduction (p < 0.001) in PPD scores. Both ASHAs and mothers had high enthusiasm for the intervention methods and activities.

**Discussion:**

This ASHA-delivered BA intervention was found to be feasible, acceptable, and effective in treating PPD in rural Indian mothers. This corroborates literature that demonstrates the efficacy of a BA intervention among individuals with generalized depression in South Asia. In communities with minimal mental health resources, interventions led by trained community workers have the potential to address PPD.

## Introduction

Postpartum depression (PPD) is a worldwide phenomenon, with women from all demographic, cultural, and religious groups affected. Estimates suggest that globally 17% of women experience PPD, with low- and middle-income countries (LMICs) reporting a higher prevalence than high-income countries (1). These disproportionate rates are concerning, as many LMICs lack the resources necessary for screening, identification, and treatment of mental illness. In India, a meta-analysis estimated a 22% PPD prevalence rate, with results as high as 31% in South India (2). While genetic history and medical history are contributory factors, social environment also plays a role in PPD. In India, for example, preference for a male child, domestic violence, and economic difficulties are contributing factors, as well as local practices for postpartum mothers such as limiting food and drink and restrictions on leaving the house or socializing for several months (3, 4).

Defined as part of a more comprehensive diagnosis that includes depressive symptoms emerging during pregnancy or after childbirth, PPD symptoms and illness trajectory vary. When untreated, 30%–50% of women will experience ongoing symptoms as long as 1 year postpartum (5). Maternal consequences may include poor quality of life, impaired social and family relationships, risky behaviors such as substance use, and, in extreme cases, completed suicide. Infant consequences include impaired maternal bonding, breastfeeding complications, difficulty with weight gain, developmental delays, and risk for maternal maltreatment (6). Proactive screening and effective treatment are essential to decrease the risk of adverse outcomes for both mother and child.

Behavioral activation (BA), an individual-level treatment for depression, was developed by Lewison and colleagues in the, 1970s (7). BA assists depressed individuals with reengagement in their lives through focused activities that address patterns of avoidance, withdrawal, and inactivity. The program was designed to help individuals with engagement and positive reinforcement to counter depression (8). Randomized controlled trials in India have demonstrated that psychosocial interventions such as BA developed in high-income countries can be modified and delivered by non-physician health workers in LMICs (9). For example, a lay counselor-delivered BA intervention with 495 community-dwelling adults (75% of whom were women) diagnosed with moderately severe to severe depression in Goa, India, was found to significantly lower symptom severity when compared to usual care (10).

Recent reductions in maternal mortality provide the opportunity to shift focus to address maternal mental health problems including PPD (11). The Indian Mental Healthcare Act of, 2017 outlined objectives to increase universal access to mental health care, congruent with the United Nations’ Sustainable Development Goals (Goal 3-Health) (11). While a welcomed policy, continued barriers exist to implementing feasible, sustainable, broad-reaching initiatives to improve maternal mental health outcomes. Access to mental health services is one barrier, with a widely reported ratio as low as 0.75 psychiatrists per population of 100,000 (12). Complex help-seeking patterns, the result of culturally derived norms, including stigma, misconceptions, and knowledge deficits related to mental illness, all limit care-seeking behaviors (13). Stigma and negative attitudes toward patients with mental illness are also documented among healthcare workers (14). Issues specific to pregnancy and PPD include the lack of screening protocols as part of the standard of care, hesitancy to treat PPD with pharmacological modalities, minimal ability to refer, and maternal stigma, all of which necessitate an innovative approach to timely and effective identification and treatment (15).

Community health workers (CHWs), known as accredited social health activists (ASHAs), are trained as part of India’s National Rural Health Mission and are equivalent to CHWs found globally. ASHAs serve as gatekeepers for maternal health initiatives, having familiarity and rapport with women in their community (16). ASHAs conduct routine visits with women in both the prenatal and postnatal periods as part of usual care delivery. ASHA maternal–child health training content is broad and provides only minimal information about mental health in general or about PPD specifically. Integration of ASHAs for screening and delivering BA for women with PPD may serve as a strategy to optimize health outcomes among postpartum women, addressing barriers in India and other LMICs.

The two research questions guiding this study were as follows:

- What are the feasibility and acceptability of ASHAs screening for and delivering a brief behavioral activation intervention addressing PPD among women in Belagavi, South India?
- What impact did the brief behavioral activation intervention have on PPD?

## Methods

We implemented a single-arm pilot study using mixed quantitative and qualitative measures to explore the feasibility of using ASHAs to screen for and deliver a brief behavioral intervention aimed at treating PPD and to assess the acceptability of the intervention among women testing positive for postpartum depression.

We obtained approval for this study from the Thomas Jefferson University Institutional Review Board, the Institutional Ethics Committee of the KLE Academy of Higher Education and Research (Ref: 70121010), and from the Karnataka State Government Department of Health and Family Welfare, which oversees ASHA role responsibilities. The interprofessional research team from both US- and India-based academic institutions included a nurse midwife, a psychiatrist, a public health nurse-researcher, a psychiatric nurse practitioner, and mental health and community nursing faculty.

### Training

A total of 17 ASHAs working locally were recruited to participate in a 4-day training on the campus of an India-based affiliated university. ASHAs were compensated for their transportation to the university and for their time in training. Permission for their participation was obtained from local supervisors. Training materials were developed by the research team, based on a review of the literature and findings from a qualitative study of local stakeholders (4). Content included an oral assessment of perceptions, knowledge, and experiences related to PPD using interactive lectures, videos, role-playing, and storytelling. Details about the study protocol, the ASHAs’ role in the study, therapeutic communication methods, and the responsible conduct of research were also presented. All training materials were designed for the education and literacy levels of the ASHAs.

### Recruitment

Eligibility criteria for potential participants included women who a) were 6 – 12 weeks postpartum, b) had no self-reported history of previous substance use disorder or psychiatric illness, c) were 18 years of age or older, and d) could read and write in the local dialects of Kannada or Marathi and screened positive for mild to moderate PPD. Exclusion criteria were 1) women who answered affirmatively to a question indicating thoughts of self-harm) and 2) women with severe depression (scores on the screening instrument of more than 14). Women with either of these exclusion criteria were referred to the team psychiatrist.

To identify an appropriate sample, researchers and ASHAs used local primary health centers. These centers are the main entry point for primary health services throughout India; each is responsible for the care of approximately 30,000 Indians. The researchers and ASHAs together attended 38 pediatric vaccination clinic sessions between May and November, 2022 at seven primary health care (PHC) clinics in Belagavi, Karnataka, India. Women who met eligibility criteria and indicated an interest in screening worked with researchers and ASHAs to review the informed consent document. To ensure ASHAs’ comfort in completing these tasks, researchers initially assisted the ASHAs and then gradually assumed a more passive role, available for questions.

### Intervention

ASHAs visited postpartum women enrolled in the study for five weekly visits. At each visit, the ASHAs asked the women to select five activities that they would do daily from a list of culturally appealing activities such as cooking a new recipe, having a 10-minute phone conversation with a friend, or knitting a hat for the baby. At subsequent visits, ASHAs reviewed the activities in which the women had participated, made goals for the upcoming week, and assessed progress from the previous week using a weekly activity log. At each visit, ASHAs also asked if participants had any thoughts of self-harm or harm to others. Each ASHA was assigned a designated member of the research team who was readily available by phone, should participants answer in the affirmative. Researchers made random visits with the ASHAs to ensure adherence to the study protocol.

### Data collection instruments

To screen for PPD and identify women whose screening indicated the presence of depressive symptoms, we used the Kannada language version of the Edinburgh Postnatal Depression Scale (EPDS). Screening with the EPDS is standard of practice in the USA and is commonly used in many LMICs (17). The scales’ 10 items ask frequency of symptoms (e.g., “I feel anxious or worried for no good reason; I have been so unhappy that I have had difficulty sleeping”) with four levels of responses (“not at all; not very often; sometimes; and yes, most of the time”). Possible scores range from 0 to 30, with scores of 10 or greater indicating minor or major depression. The validity of a Kannada-language version of the EPDS has been demonstrated in past research.

Intervention feasibility for participants was assessed by self-report and completion of intended and actual BA activities in the adapted Participant Weekly Behavioral Activity Log. Acceptability was assessed using the Behavioral Activation Intervention Acceptability Survey (BAIAS) (18), an eight-item (one open-ended and seven Likert-scored questions) instrument for ASHAs by the System Usability Scale (19). Higher scores on each of these instruments suggest greater feasibility and acceptability of the intervention. Open-ended individual and group interviews with both participants and ASHAs also addressed both feasibility and acceptability.

All instruments were translated into the local language of Kannada by researchers who have previously provided this work, back-translated to ensure accuracy, and pilot-tested before program implementation.

Feasibility and acceptability were also assessed 1 month after completion of the intervention with two focus group interviews with ASHAs to capture their perspectives about their role as PPD screeners and as BA interventionists.

### Data collection

ASHAs collected the baseline screening information from postpartum women using the EPDS at vaccination clinics. Researchers were present during screening to ensure adherence to the screening protocol and provide feedback as needed. For women who screened positive and participated in the intervention, ASHAs collected a follow-up EPDS score at women’s homes at the final visit to assess intervention efficacy. Members of the research team conducted individual interviews with each participant following the 5-week intervention. To assess ASHAs’ perspectives on the feasibility of PPD screening and intervention implementation, ASHAs participated in a focus group 2 weeks after the completion of the final participant visit. All interviews were held in a private room at the local PHC.

Multiple attempts were made to ensure the privacy of data collection, with mothers invited to be interviewed in separate rooms or outdoor areas. In a few cases, the interview was conducted in the presence of the patient’s mother.

### Data analysis

Quantitative data were entered into a REDCAP database by one member of the research team (VN) and transferred to a SAS 9.4 program (20). Before conducting the pre-test/post-test analysis, we created a difference variable by subtracting the pre-PPD score from the post-PPD score for each participant. We generated a paired t-test to compare the pre-assessment with post-assessment results for PPD score. For this method of analysis to be valid, we assumed that the data were symmetrically distributed around the mean. Using the univariate procedure in SAS, we checked for significant violations of the normal assumptions. In addition, we performed Wilcoxon signed rank tests, which require no parametric assumption. For the categorical predictors with small cell sizes, we also considered the non-parametric Wilcoxon rank sum test. A significance level was set at the alpha = 0.05 level.

Researchers transcribed the qualitative data verbatim from audio recordings and translated them into English. We analyzed transcriptions using NVivo software version, 13 (21). Two independent investigators (AS and PK) experienced in qualitative research open-coded the transcripts. We reviewed codes with the principal investigator/author to resolve any discrepancies. The research team identified major themes and selected relevant quotes. To ensure trustworthiness, credibility, transferability, dependability, and confirmability, researchers adhered to the tenets of Lincoln and Guba (22).

## Results

Over the screening period, we screened 83 postpartum women. One woman disclosed thoughts of self-harm and was immediately referred to the psychiatrist researcher who offered treatment at the affiliated tertiary care institution. Twenty-six of the 83 women (31%) screened positive for PPD. There were no demographic differences between women who did and did not screen positive. No eligible women declined screening. After two of the 26 moved, 24 women participated in and completed the intervention.

Of the 24 women who completed the intervention, the average age was 27 years (range 20–35, SD 4.9); 15 (62.5%) had an elementary education, and 10 (38.9%) had secondary school education; 21 (87.5%) were Hindu, and three (12.55%) were Muslim. This was a first child for 10 (41.7%) women. These and other descriptors are presented in **Table 1**.

**Table 1:** Demographics of Sample.

Education Primary High school Graduate	15/62.55% 8/33.3% 1/ 4.2%
Number of chidren One Two >Two	10/41.7% 7/29.2% 7/29.2%
Sex of Baby Female Male	14/58.3% 10/41.7%
Religion Hindu Muslim	21/87.5% 3/12.5%
Planned pregnancy Yes	15/62.5%
Baby complications Yes	2/8.3%
Maternal complications Yes	5/20.8%
Mean EPDS scores at baseline	11.75 ±1.22

All participants had highly significant changes (p < 0.001) in PPD scores (mean change −10.63, SD 1.44). Similar results were obtained by both the non-parametric Wilcoxon signed rank test and the paired t-test.

BAIAS showed high acceptability with an average of 4.32 (SD 0.89). All 24 participants completed the activities on their weekly Behavioral Activity Log, also suggesting high acceptability. Qualitative results from ASHAs and participants suggested enthusiasm for the training and intervention components.

From the ASHAs:

*ASHA #3: Initially, we were concerned about administration of the EPDS. This was resolved during training by performing role play based on various scenarios from the training officers and later [by working with] the participants.*

*ASHA #5: After training, we were much more confident in screening through the EPDS and [doing] the intervention through role play.*

*ASHA #8: The tool [EDPS] was in the local language which made it easy to screen the mothers.*

*ASHA #2: All ASHA workers need to be trained. These Activities were non-pharmacological and don’t involve any risk.*

*ASHA #10: If more ASHAs were trained … it is helpful in preventing PPD by regular BA activities.*

From the mothers:

*Mother #9: I felt confident day by day doing the BA, as these activities were simple and was able to complete the tasks each day. This boosted my self-confidence.*

*Mother #8: It was a good experience. It strengthened my personality and my mood.*

*Mother #19: Activities were so simple and free from hassle.*

*Mother #2: I encountered no such problems [when engaging in BAI activities]. All activities were so simple and easy to perform at home along with baby care.*

## Discussion

The results of this pilot study suggest that community health worker-administered behavioral activation interventions have the potential to address PPD in settings with minimal mental health resources.

The very high levels of acceptability and completion of activities among participants were surprising. While it is not possible to discount the social acceptability of responses to questions, that is, that ASHAs and participants stated what they thought the research team wanted to hear, it is likewise possible that the BA model is actually very patient-friendly and feasible to implement. This latter possibility is supported by the findings in a recent community-based Randomized controlled trial in which 83.9% of participants in the BA arm completed their assignments (23). Similarly, studies of BA with cancer patients found completion rates of 76.2% and 77.3% (24, 25).

The surprisingly high change in scores of all participants on the EPDS suggests either a highly effective community-based intervention or the negative feelings and mood swings experienced by many new mothers are transient as the result of hormonal changes and sleep deprivation. While our positive screening rate of 31% was consistent with that found by other researchers in southern India (2), a randomized controlled trial with a larger sample size and serial assessments of the mental health status of new mothers is necessary to understand the natural history of PPD and the role of community-based interventions to address this problem.

Our positive feasibility and acceptability results provide preliminary evidence that the ASHA role can be expanded to include screening for PPD. ASHAs have familiarity with women in their communities, and their current workload involves routine visits with women in both the prenatal and postnatal periods. They also have positive attitudes about community mental health issues and have worked to expand mental health services in selected communities during the COVID-19 pandemic (26). However, such role expansion must be coupled with adequate training, supervision, and compensation to be scalable and sustainable (16).

This study had several limitations. We did not have a mechanism to confirm women’s participation in the daily BA activities; however, the ASHAs had trusted interpersonal relationships with the women with whom they work, and participants would feel comfortable disclosing non-adherence. The lack of a control group that would provide information about the natural history of PPD means that we cannot state that the intervention was responsible for changes in depression scores. Though the lack of privacy might have affected the validity of some cases, the results are still generalizable to the Indian population where privacy in clinical settings is still not a regular practice. The small sample made an effect size difficult to calculate.

This community health worker-mediated behavioral activation intervention was found to be feasible and acceptable in treating PPD in rural Indian mothers. Large-scale, controlled studies are needed to confirm these findings.

## Data availability statement

The raw data supporting the conclusions of this article will be made available by the authors, without undue reservation.

## Ethics statement

The studies involving humans were approved by Thomas Jefferson University IRB and KLE University Ethics Review Board. The studies were conducted in accordance with the local legislation and institutional requirements. The participants provided their written informed consent to participate in this study.

## Author contributions

AS: Funding acquisition, Writing – original draft, Writing – review & editing. BT: Funding acquisition, Writing – original draft, Writing – review & editing. VN: Writing – original draft, Writing – review & editing. GU: Writing – original draft, Writing – review & editing. MS: Writing – original draft, Writing – review & editing. SD: Writing – original draft, Writing – review & editing. UK: Writing – original draft, Writing – review & editing. SP: Writing – original draft, Writing – review & editing. SR: Writing – original draft, Writing – review & editing. VS: Supervision, Writing – review & editing. PK: Funding acquisition, Writing – original draft, Writing – review & editing.
